# Supplementary material for: Clinical & laboratory profiles and treatment outcome of Kawasaki disease in children: Experience from a tertiary care hospital
Source: PLoS One. 2025 Oct 3;20(10):e0328443. doi: 10.1371/journal.pone.0328443 (PMC12494243; doi:10.1371/journal.pone.0328443)
Supplement: Attachment — Submitted filename: Response to Reviewers_PONE-D-24-55276.docx [file pone.0328443.s002.docx]

**Response to Reviewers**

**Responses to the comments of the academic editor**

**Comments to the Author**

1. Is the manuscript technically sound, and do the data support the conclusions?

The manuscript must describe a technically sound piece of scientific research with data that supports the conclusions. Experiments must have been conducted rigorously, with appropriate controls, replication, and sample sizes. The conclusions must be drawn appropriately based on the data presented.

Reviewer #1: Partly

Reviewer #2: Yes

**Response**: We have now revised the manuscript to make this technically sound and the data now support the conclusion

2. Has the statistical analysis been performed appropriately and rigorously?

Reviewer #1: No

Reviewer #2: Yes

**Response**: Thanks. We have revised the statistical analysis section to make this more appropriate and rigorous

3. Have the authors made all data underlying the findings in their manuscript fully available?

The [PLOS Data policy](http://track.editorialmanager.com/CL0/http:%2F%2Fwww.plosone.org%2Fstatic%2Fpolicies.action%23sharing/1/010f0196c724c80d-6cbe3f4e-7db6-4944-a79a-a368e6bf5e79-000000/oj77n8qN4ezSd9x1RZYh18Bass0pYTAnedKJeRgsey4=210) requires authors to make all data underlying the findings described in their manuscript fully available without restriction, with rare exception (please refer to the Data Availability Statement in the manuscript PDF file). The data should be provided as part of the manuscript or its supporting information, or deposited to a public repository. For example, in addition to summary statistics, the data points behind means, medians and variance measures should be available. If there are restrictions on publicly sharing data—e.g. participant privacy or use of data from a third party—those must be specified.

Reviewer #1: Yes

Reviewer #2: Yes

**Response**: Thanks.

4. Is the manuscript presented in an intelligible fashion and written in standard English?

PLOS ONE does not copyedit accepted manuscripts, so the language in submitted articles must be clear, correct, and unambiguous. Any typographical or grammatical errors should be corrected at revision, so please note any specific errors here.

Reviewer #1: Yes

Reviewer #2: Yes

**Response**: Thanks.

5. Review Comments to the Author

Please use the space provided to explain your answers to the questions above. You may also include additional comments for the author, including concerns about dual publication, research ethics, or publication ethics. (Please upload your review as an attachment if it exceeds 20,000 characters)

Reviewer #1: This manuscript examines Kawasaki Disease in children at a Bangladeshi tertiary care hospital, providing valuable data through detailed clinical and laboratory profiles of 40 cases. While the study's strengths include clear documentation of treatment outcomes, several concerns need addressing - notably the unusually high rates of incomplete KD and coronary abnormalities, statistical rigor, methodology clarity, and limited discussion of findings in context of current literature. With appropriate revisions, this work could meaningfully contribute to understanding KD in Bangladesh.

**Response**: Thanks for your constructive review comments. We have revised the manuscript following your kind suggestions.

Major Comments:
1. While several laboratory tests were performed (CBC, ESR, CRP, SGPT, S. creatinine, blood C/S, Urine R/ME, C/S), the Results section only presents and analyzes a limited subset of these findings, primarily focusing on CRP, ESR, and CBC parameters. The data from other tests like SGPT, creatinine, blood culture, and urinalysis are mentioned in Methods but not reported or analyzed in the Results. To maintain scientific rigor and completeness, either all laboratory data should be presented and analyzed to justify the term "laboratory profiles" in the title, or the title should be modified to reflect the actual scope of reported findings.

**Response**: Thanks. We have provided the information of all mentioned investigations in the results (page 6 under table 1).

2. Your introduction requires updating with literature from 2022-2024 to better contextualize your findings within current Kawasaki Disease research. We recommend incorporating recent epidemiological data (especially from South Asia), modern diagnostic approaches, and contemporary treatment outcomes. While retaining seminal papers, please replace pre-2015 references with current evidence on regional epidemiology, diagnostic criteria, and treatment guidelines to strengthen your manuscript's relevance and contribution to the field.

**Response**: Thanks. We have revised the introduction section according to your kind suggestions on pages 3 & 3 under introduction.

3. Clarify diagnostic criteria used for incomplete KD.

**Response**: Thanks. It is now mentioned on pages 4 and 5 under section material and methods section.

4. The statistical methodology requires significant revision. While P-values are presented, the specific statistical tests used are not specified, and there is no sample size calculation or power analysis. The authors should clearly state which statistical tests were employed and provide justification for the sample size to ensure scientific validity.

**Response**: Thanks. This is an exploratory study where we enrolled all the children (n=40) diagnosed with KD between June 2019 to April 2023. However, we have performed the sample size calculation which has been mentioned on page under sample size on page 5. We have also done the power analysis of the study which is discussed on page 14, prior conclusion, under discussion section. Moreover, we have revised the statistical analysis plan according to the suggestion of the respected reviewer.

5. Add multivariate analysis of risk factors for coronary involvement.

**Response**: Thanks. We have introduced multiple logistic regression and mentioned this in the statistical analysis plan on page 6 (first paragraph) and results section (table V, page 10 on results section)

6. Figure 1 is overly simple - additional figures showing key findings would be valuable.

**Response**: Thanks. We have deleted this figure as this information is available in the results on page 6.

7. The Discussion section needs substantial revision as it currently focuses too heavily on restating results rather than interpretation and context. We recommend reducing the descriptive content of findings and expanding the analytical discussion, particularly comparing your results with contemporary literature and explaining the high rates of incomplete KD and coronary involvement observed in your study. Include more critical analysis of how your findings contribute to current understanding of Kawasaki Disease in South Asian populations.

**Response**: Thanks. We have revised discussion section on pages 11 to 13 addressing the above-mentioned suggestions.

8. The authors should strengthen the discussion by citing contemporary literature to explain why their study found unusually high rates of incomplete Kawasaki Disease (65%) and coronary involvement (95%) compared to global data, with particular focus on whether these findings reflect delayed diagnosis, regional genetic factors, or referral bias in their tertiary care setting.

**Response**: Thanks. We have done this accordingly throughout the manuscript and also in the reference list.

Reviewer #2: 1. The dose of Aspirin in the acute phase may also affect the coronary artery on follow-up. It needs to be kept under consideration and can be included if it varies.

**Response**: Thanks. We concur with the concern of the respected reviewer and. It is important to mention that we have used adequate dose of aspirin and mentioned in table III.
2. The article is an addition to the previous studies where atypical Kawasaki is increasing now.

**Response**: Many thanks for your positive comments.
